# An evaluation of childhood tuberculosis program in Chegutu District, Zimbabwe, 2020: a descriptive cross-sectional study

**DOI:** 10.1186/s12913-022-07918-2

**Published:** 2022-04-14

**Authors:** Memory Chimsimbe, Pride Mucheto, Tsitsi Patience Juru, Addmore Chadambuka, Emmanuel Govha, Notion Tafara Gombe, Mufuta Tshimanga

**Affiliations:** 1 Department of Primary Health Care Sciences, University of Zimbabwe, Harare, Zimbabwe; 2 Department of Oral Health, University of Zimbabwe, Harare, Zimbabwe; 3 Zimbabwe Field Epidemiology Training Program, 3-68 Kaguvi Building, Corner 4th/Central Avenue, Harare, Zimbabwe; 4 African Field Epidemiology Network, Harare, Zimbabwe

**Keywords:** Childhood tuberculosis, Program evaluation, Zimbabwe

## Abstract

**Background:**

Childhood tuberculosis (TB) is a major global public health concern contributing to significant child morbidity and mortality. A records review of the TB notification for Chegutu District Health Information System 2 (DHIS2) showed a low childhood TB case detection rate. For 2018 and 2019, childhood TB notifications were 4% and 7% respectively against the annual national childhood 12% case detection rate. We evaluated the performance of the childhood TB program in Chegutu.

**Methods:**

We conducted a descriptive cross-sectional study. Sixty-six health workers (HW) participated in the study. Interviewer-administered questionnaires and checklists were used to collect data on reasons for low TB case detection, HW childhood TB knowledge, program inputs, processes, and outputs. Strengths, Weaknesses, Opportunities and Threats analysis was used to assess the childhood TB processes. We analyzed the data using Epi Info 7™ to generate frequencies, proportions and means. A Likert scale was used to assess health worker knowledge.

**Results:**

The majority 51/66(77%) of HW were nurses and 51/66(67%) of respondents were females. Reasons for the low childhood TB case detection were lack of HW confidence in collecting gastric aspirates 55/66(83%) and HW’s negative attitudes towards gastric aspirate collection 23/66(35%). HW 24/66 (37%) had a fair knowledge of childhood TB notification. The district had only one functional X-ray machine for 34 health facilities. Only 6/18 motorcycles were functional with inadequate fuel supply. No desk guide for the management of TB in children for HW (2018) was available in 34 health facilities. Ethambutol 400 mg was out of stock and adult 800 mg tablets were used. Funds allocated for motor vehicle and motorcycles service ($1612USD/year) were inadequate. The district failed to perform planned quarterly TB review meetings, contact tracing and childhood TB training due to funding and COVID-19 lockdown restrictions.

**Conclusion:**

The childhood TB program failed to meet its targets due to inadequate inputs, HW suboptimal knowledge and COVID-19 lockdown measures. Case detection and notification can be improved through on-job training, mentorship, support and supervision and adequate resources.

## Background

Childhood tuberculosis (TB) is a major public health problem that contributes to significant morbidity and mortality in children [1]. Historically, tuberculosis has been neglected in children. The neglect can be explained by the difficulties in confirming a diagnosis of TB in children and the fact that children are not included in most surveys, poor recording and reporting practices, and misjudgement of tuberculosis in children as a low public health priority [2].

Childhood tuberculosis notifications can be compared to total tuberculosis notifications to determine what proportion of TB cases are from children. The number of people who were diagnosed with TB and were notified should be more than 90% of all incidence cases in the same year. Globally, children account for approximately 10–20% of all TB notifications [3]. Twenty-five per cent of the ten million people who were diagnosed with TB in 2019 were from the African region, with 10–20% of all TB cases expected to have occurred in children [3]. In Zimbabwe, 25,775 people were diagnosed with TB in 2019, and about 10% of these were children below 15 years [4, 5]. Despite TB being curable and preventable, nearly 650 children die from TB every day, with 80% of them dying before their fifth birthday [3].

Zimbabwe is a high TB burden country [3], and childhood TB notification is less than 10% annually with notification trends continuing on a downward spiral [5]. The Government of Zimbabwe in its commitment to the control of TB adopted the Directly Observed Treatment, Short-course (DOTS) Strategy in 1994. Zimbabwe subscribes to the World Health Organisation (WHO) End TB strategy of reducing TB incidence by 90% by 2035 [5]. A Desk Guide for Management of TB in children for health care workers was developed to improve TB management in children [6]. Ministry of Health and Child Care (MOHCC) developed the Zimbabwe National Strategic TB Plan 2017–2020. One of the strategic objectives the nation is pursuing is; to increase the case detection and treatment of all forms of TB from 72% in 2015 to 85%, with contribution from childhood TB increasing from 7 to 12% by 2020 [5].

A review of Chegutu District Health Information System (DHIS2) TB notification records showed that childhood tuberculosis case detection rate was low, in 2018 and 2019 tuberculosis notifications in children were 4% and 7% respectively against the annual national childhood TB case detection target of 12%.

Low case detection may imply that TB cases in children are missed, tuberculosis transmission and new infections will continue to rise unnoticed leading to childhood morbidity and mortality. The childhood TB notification in children of 12% in 2020 will not be met. The district was supported by Biomedical Research and Training Institute (BRTI**)** through the collection of sputum specimens from each facility twice weekly. Health care worker training on TB was done in 2018, 2019 and 2020. Ongoing TB mentorship, support and supervision were done in the district quarterly. Despite these efforts, childhood TB case detection remained low. There is no documented evidence to show that the childhood program in Chegutu was evaluated to assess its performance. We, therefore, evaluated the childhood tuberculosis program in the Chegutu District to make evidence-based recommendations to help improve the program.

## Methods

### Study design

We conducted a descriptive cross-sectional study based on the Centre for Disease Control and Prevention (CDC) logic model for program evaluation [7].

### Study setting

The study was conducted in Chegutu District, Mashonaland West Province situated in the central northern region of Zimbabwe from March 2021 to May 2021 whilst evaluating the childhood TB program in 2020. The district has 34 health facilities including a district hospital. It has a total population of 180,741 people [8]. Rural residents travel ten to twenty-five kilometres to access health care facilities. All government, rural and urban councils’ health facilities were participating in the childhood TB program. The economic activities in the district consist of indigenous companies, mining, commercial farming and subsistence farming.

### Study population

Our study population were health care workers from the outpatients’ department, maternal child health department, pediatric wards, laboratory, pharmacy department and environmental health department. The Environmental Health Technicians (EHT) are responsible for transporting TB specimens from the health facilities to the two TB diagnosing centres in the district as well as sending TB results to the health facilities. Moreso, EHTs conduct TB contact tracing as well as provision of health education on infection prevention and control in the community. Caregivers of children being treated for TB in 2021 were interviewed. The District Medical Officer, the District Nursing Officer, Acting District TB Coordinator, District Environmental Health Officer, Senior Nursing Officer, District Pharmacist, Logistic Officer National Pharmaceutical Company, and Laboratory Scientist were key informants in the study.

The TB presumptive register captures information for all patients screened for TB. The information includes demographic data; TB risk group such as being under five, malnourished or HIV positive; type of TB specimen collected; date Chest X-ray was taken and HIV result. The TB register captures all TB cases detected. It contains the following information: date of diagnosis, date of notification, demographic data, TB risk group; TB laboratory results; follow updates; TB/HIV care, contacts screened; TB medicines supply dates and treatment outcome. The TB notification forms, presumptive register and TB registers were reviewed for TB screening done, TB treatments done, TB contacts screened, and TB notifications.

Laboratory stock cards were checked for monthly stocks of TB consumables kits. This kit provides a convenient way to receive all of the needed reagents and consumables to perform 1000 smears using bright field Ziehl Neelsen (ZN) microscopy. Laboratory stock cards were checked for monthly stocks of Gene Xpert cartridges. Pharmacy stock cards were checked for stocks of childhood TB medicines.

### Sample size for health care workers

We calculated a sample size of 66 health care workers using the Dobson formula:

*n* = z_a_^2^ x p (1-p)/ delta^2.^, assuming that 96% of the health workers knew that TB is curable from a study by Pantha et al. (2020) in Bangladesh [9] at a 95% confidence interval (CI), 80% power, a margin of error of 5% and a non-response rate of 10%).

### Sample size for caregivers of children with TB

There were five caregivers with children who were still on TB treatment as the other children 16 children who were commenced on TB treatment in 2020 had completed their 6 months. We conveniently recruited the five caregivers into the study.

### Sampling of health care workers

Health care workers from sixteen high volume sites and three hospitals in the district were recruited into the study. The 31 clinics in the Chegutu District have a staff complement of three nurses at each clinic. On the day of data collection, where three nurses reported for work, simple random sampling using a random number generated by the RANDBETWEEN function in Microsoft Excel was used to select two. If only one nurse reported for work, we recruited him or her into the study. At hospitals (Chegutu District, Mhondoro Rural and Norton) we randomly selected 12 health care workers from (outpatients department, opportunistic infection clinic, maternal child health department, paediatric ward, laboratory, pharmacy department and environmental health department) using random numbers generated by the RANDBETWEEN function in Microsoft Excel. Key informants were purposively recruited into the study.

### Data collection

#### Health care workers

We collected data from health care workers using a pre-tested interviewer-administered questionnaire on demographic information, reasons for low childhood case detection, knowledge on childhood TB and processes involved in childhood TB. Presumptive registers, TB registers, notification forms, pharmacy ordering forms, laboratory ordering forms and stock cards were reviewed to assess for childhood TB program outputs and outcome indicators. A checklist was used to assess childhood TB program resources availability.

#### Key informants

A key informant guide was used to collect data from the key informants on the childhood TB program budget, health care worker training that was conducted, availability of resources and performance of the program in 2020. We used information collected from key informants to triangulate quantitative findings from health care workers, caregivers and childhood TB records.

#### Caregivers of children with TB

We used a pretested questionnaire to collect information from caregivers on their views regarding childhood TB.

### Data analysis

We used Epi Info™ 7 statistical software to capture and analyze data. Descriptive statistics were used to describe the study population and were presented as frequencies, proportions and median. Knowledge of health care workers was assessed using a 3-point Likert scale (good, fair, poor). We used five questions and one mark was awarded for a correct answer to any of the five questions. Four or five marks were considered good knowledge, three marks were fair knowledge, and less than two marks was poor knowledge.

An in-depth assessment of the TB program processes *(childhood TB case finding, TB notification process TB contact tracing, the procurement and distribution process of childhood TB medicines and laboratory consumables)* was done using the Strengths Weaknesses Opportunities and Threats (SWOT) analysis. Qualitative data from key informants were grouped manually into themes and then analysed by theme.

## Results

We interviewed sixty-six health care workers and five caregivers of children on TB treatment and the response rate was 100%. The health care workers included nurses, doctors, environmental health technicians, pharmacy technicians, microscopists, and laboratory technicians.

### Demographic characteristics of caregivers and health workers in childhood TB program in Chegutu District, 2020

The majority 51/66 (77%) of health care workers were nurses. Seventy-seven (51/66) of the respondents were females. The median age of health care workers was 39 years (IQR 31–57) and the median years in service of health care workers was 10 years (IQR 4–14). The median age of caregivers was 44 years (IQR 40–52) and the median age of children on TB treatment was 12 years (IQR 4–14). Four out of the five children on TB treatment were males **(**Table 1**).**

**Table 1:** Demographic characteristics of health workers and caregivers in childhood TB program, Chegutu District, 2020

Variable	Category	Frequency (*n* = 66)	Percentage
Sex	Female	51	77
	Male	15	23
Age		Median age = 39 years IQR 31–57	
Designation	Nurses	51	77
	Environmental Health Technician	5	8
	Doctors	4	6
	Laboratory Technician	3	5
	Pharmacy Technicians	2	3
	Microscopist	1	1
Years in service		Median: 10 years IQR 4–14	
Caregivers		5	
Sex of child	Male	4	
	Female	1	
Age of child in years		Median: 12 years IQR 4–14	

### Reasons for low childhood TB case detection

The reasons for the low childhood TB case detection reported by health care workers were lack of health care worker confidence in collecting gastric aspirates 55/66 (83%), high workloads in health facilities 27/66 (41%), health care worker’s negative attitudes towards gastric aspirate collection (feeling uncomfortable when inserting a nasogastric tube, and the procedure is painful for the child) 23/66 (35%) and health care worker lack of knowledge on childhood TB 35/66 (35%).

### Health care worker knowledge assessment on childhood TB program

On knowledge assessment, most of the respondents 49/66 (74%) knew that swollen lymph node was a common manifestation of extrapulmonary TB in children. Seventy per cent 46 /66 knew more than three key risk factors for TB in children which included the following: household or other close contacts with a case of smear-positive pulmonary TB; age less than f5 years; HIV infection; severe malnutrition and recent measles infection. Sixty-seven per cent (44/66) of the respondents knew more than three investigations that are carried out for children who could not produce sputum which included the following: stool for gene Xpert; Tuberculin Skin Test; Chest X-ray; gastric aspirate for gene Xpert; biopsy for microscopy and histology and fine needle aspirate for microscopy and histology. Fifty-five per cent (36/66) of the respondents knew three or more symptoms of TB in children less than 10 years. Thirty per cent (20/66) of the respondents knew that enlarged lymph nodes for suspected TB in children were two centimetres more in diameter. Using the 3-point Likert scale, healthcare workers had fair knowledge 24/66 (37%) **(**Table 2**).**

**Table 2:** Health worker knowledge assessment of the childhood TB program, Chegutu District, 2020

Variable	Frequency *n* = 66	Percentage
Know that lymph node was a common manifestation of Extrapulmonary TB in children	49	74
Know the key risk factors for TB in children	46	70
Know the investigations done for children who cannot produce sputum	44	67
Know the main symptoms of pulmonary childhood TB in children less than 10 years	36	55

3- point Likert scale (Good, fair, poor)		
Fair	24	37
Good	22	33
Poor	20	30

### Caregivers’ views of childhood TB program

We interviewed five caregivers of children on TB treatment about their experiences in the process of the diagnosis and treatment of TB. One (1/5) caregiver highlighted that TB was diagnosed after two weeks of illness due to a lack of funds for a chest x-ray. Three (3/5) of the children had sputum collected for Gene Xpert within 24 hours of being a presumptive case. No medicine stock-outs were reported by caregivers, however, one (1/5) caregiver highlighted challenges with breaking Ethambutol tablets into two due to a shortage of pediatric TB formulations.

### Inputs injected into the childhood TB program in Chegutu District, 2020

There were two functional Gene Xpert machines and one functional X-ray machine in the district for 34 health facilities. There was one non-functional motor vehicle for acquired immunodeficiency syndrome (AIDS) and TB program without fuel allocation to assist in the childhood TB program. There were three motorcycles from the Biomedical Research and Training Institute (BRTI**)** that supported the collection of sputum specimens from each facility twice weekly. The funds allocated for servicing motor vehicles and motorcycles were inadequate **(**Table 3**).**

**Table 3:** Inputs injected into the childhood TB program, Chegutu District, 2020

Item	Available	Expected	Comment
Funds for TB vehicle servicing	$806USD /year	$4000USD/year	Funds were not adequate for servicing; money was only used for repairs
Funds for motorcycle servicing	$806USD /year	$4000USD/year	Funds were not adequate for servicing; money was only used for repairs
Fuel	20 litres /week for a bike	40 litres a week for a bike	Inadequate fuel for specimen collection and contact tracing.
Vehicle	1	2	The vehicle was non-functional in 2020
Motorcycles	18	1/ health facility =34	Only six motorcycles were functional in 2020
TB consumable kit	12kits	12kits	Adequate
Gene Xpert cartridges	15 × 50 cartridges	15 × 50 cartridges	Adequate
Rifampicin/Isoniazid/Pyrazinamide	10 tins ×84 tablets (above minimum stock)	12tins × 84 tablets	Adequate
Rifampicin/Isoniazid	10 tins × 84 tablets (above minimum stock)	12 tins × 84 tablets	Adequate
Isoniazid 100 mg	20 tins × 100 tablets	12 tins × 100 tablets	Above maximum stock
Ethambutol 400 mg	0	2 tins × 1000 tablets	Using adult tablets
Desk Guide for the management of TB in Children for Health Workers (2018)	0	2/health facility	Not available in all health facilities.
Tuberculin skin test	10 ampoules	10 ampoules	Were adequate. Not requested from the pharmacy since March 2020.
National TB/HIV management guidelines	1/ health facility	1/health facility	Were adequate in the district
TB screening tools	4/ health facility	4/ health facility	Only adult TB screening tools were available
Gene Xpert machines	2	2	One machine had only 3 modules functional instead of 4
X-ray machine	1	2	Functional

### SWOT analysis of the childhood TB processes

#### Case finding

The district had 50 Gene Xpert cartridges per month. Gene Xpert cartridges were adequate as they were ordered monthly based on consumption. The district had nine practising doctors translating to 0.05 doctors per 1000 population which is below the WHO recommended 5.9 per 1000. Twenty two percent (30/138) of the health care workers were trained in childhood TB which enabled childhood case detection. The turnaround time for sputum positive results was 24 hours. There were 10 ampoules of Tuberculin Skin Test (TST) per quarter which were in the pharmacy since March 2020 and had never been requested for use. These were adequate based on monthly consumption of the previous quarter which was zero.

There was no Desk Guide for Management of TB in Children for Health Care Workers (2018) in the 17/17 health facilities visited.

#### Notification

All health facilities visited 17/17 had more than 20 TB notification forms per week which were adequate to notify the TB cases. Seventy-six per cent (50/66) of the health workers knew that two TB notification forms were completed during notification.

#### Contact tracing

There were more than 20 TB contact tracing forms per week in the 17/17 health facilities visited which were adequate to capture TB contacts. There are 34 practising Environmental Health Technicians in the district translating to 0.2 per 1000 population which is below the recommended WHO minimum health worker density of 4.1 per 1000 [10, 11]. All 17/17 health facilities had cell phones loaded with airtime. The district had only six functional motorbikes out of 18 as the other 12 were defective. The motorbikes were used for the collection of specimens and distribution of results from and to the sites as well as contact tracing. There was inadequate fuel allocation (20 litres /week) per motorcycle.

#### Ordering of childhood TB medicines and laboratory consumables

Two health workers per facility were trained in Zimbabwe Assisted Pull System (ZAPS) and ordering based on consumption. The order fill rate of childhood TB medicines in 17/17 health facilities was below 100%. The order fill rate of TB consumables was 100%. One health worker was hospital trained in the ordering of laboratory consumables. Ordering of laboratory consumables was based on consumption leading to no stocks out.

#### Outputs and outcomes of the childhood TB program

The proportion of notified cases was 21/539 (4%). The proportion of cases detected and started on treatment was 21/21, the proportion of cases who were tested for HIV was 21/21, the proportion of cases who were HIV positive was 14/21, the proportion of HIV cases was initiated on Antiretroviral Therapy (ART) 9/14 **(**Table 4**).**

**Table 4:** Outputs and outcomes in the childhood TB program in Chegutu District, 2020

Outputs	Achieved	Target	Comments
Proportion of notified cases	21 /539 (4%)	12%	The district did not reach its target. Children should contribute to 12% of all TB notifications.
Proportion of cases detected and started on treatment	21/21	100%	The district reached its target
Proportion of cases who were tested for HIV	21/21	100%	The district reached its target
Proportion of cases who were HIV positive	14/21		7/21 of the children were HIV negative
Proportion of HIV positive cases was initiated on Antiretroviral Therapy (ART)	9/14	100%	5/14 of the children were not put on ART as they were still within 2 weeks of commencement of TB treatment

### Results from key informants

#### Resource availability

We interviewed eight key informants involved in the childhood TB program in Chegutu District. The challenges that were highlighted by key informants included inadequate funds for motor vehicle and motorcycle servicing which led to repairs only. The district experienced fuel shortages for motorcycles, hindering contact tracing and specimen collection. Staff attrition by experienced health workers was high. The district had no funds for conducting TB training and quarterly TB review meetings which are essential in identifying gaps and strengthening the TB program. COVID-19 lockdown measures affected service delivery and TB training. The major issue from National Pharmaceuticals was the delay in the shipments of TB medicines resulting in stock-outs of some childhood TB medicines.

## Discussion

We set out to evaluate the childhood tuberculosis program in Chegutu District and determine the reasons for the low detection of tuberculosis cases in children. The reasons for the low childhood TB case detection reported by the respondents were lack of health care worker confidence in the collection of gastric aspirates and lack of knowledge on childhood TB. Lack of confidence of health care workers could be attributed to lack of knowledge in conducting gastric aspirates. Hence, health workers need to be trained to boost their confidence. Similarly, a study by Oshi et al. (2016) in Nigeria revealed that health worker knowledge, and skills in TB detection in children was low. Nasogastric or nasopharyngeal intubation for collection of specimens was not done due to the negative attitude of the procedure by health workers leading to low case detection [12].

Health workers in Chegutu District had a fair knowledge of the childhood TB signs, symptoms, investigations and risk factors for childhood TB. Fair knowledge of health workers could be attributed to lack of refresher training, mentorship and lack of supportive supervision which were disrupted by COVID-19 lockdown measures. Good knowledge plays a pivotal role in program implementation. Health care workers with good knowledge are confident in what they do. However, the health workers lacked knowledge on how to conduct gastric aspirates hence compromising their confidence to conduct the procedure. Similar findings were reported by Akma et al. (2020) in Malaysia where a knowledge gap on healthcare workers was noted on TB diagnosis resulting in low case detection [13].

The study revealed that there was inadequate funding for the childhood TB program. Limited funding resulted in poor maintenance of laboratory machinery such as the biological safety cabinet, microscopes and Gene Xpert machine, failure to repair X-ray machine, and failure to maintain a stable transportation system for sputum samples. Furthermore, the absence of vehicles for the TB control program hindered the district from conducting various activities such as delivering medicines in time or conducting supportive supervision of the health facilities involved in providing TB care. The whole district had one functional X-ray machine for 34 health facilities thereby reducing chances of clinical diagnosis of childhood TB. Resources play a key role in the success of a program. An interrupted supply of resources paralyses the program. It forms a barrier against achieving set targets. Similar findings were noted by Ereso et al. (2020) in Ethopia were inadequate resources led to low TB case finding and failure to carry out planned activities [14]. This study showed that the childhood TB program had fuel shortages as it highly depended on donor funding. This affected transportation of specimens from sites and the conducting of contact tracing Inadequate resources translate to strained processes, which in turn brings the low output of a program. TB and Leprosy Management Guidelines (2016) were available; however, the Desk-Guide for Management of TB in Children for Health Care Workers (2018) was not available in all visited health facilities. Guidelines are very important in programs as they provide step by step operating procedures for health workers. Similarly, Ereso et al. (2020) in Ethopia reported the absence of the national TB control program guideline in outpatient departments, laboratory rooms and TB rooms of the facilities leading to low case detection [14].

The processes in a program are interdependent with inputs that are injected. The process of childhood TB case detection, notification and contact tracing did not perform well in 2020. This could be attributed to COVID-19 lockdown restrictions that limited human movement [15]. The restrictions halted non-emergency medical care at health institutions and disrupted supply chains for medical consumables. Some people were also unable to access healthcare facilities due to transport issues that were caused by lockdown restrictions. Moreso, Environmental Health Technicians could not conduct contact tracing and specimen collection as expected. Lockdowns generally affected all services in the district which could explain why the TB program performance was similar to those from previous years. Similarly, Arega et al (2022) in Ethopia highlighted that COVID-19 had a negative impact on TB indicators [16].

Quarterly TB review meetings did not take place due to lack of funding and COVID-19 pandemic restrictions Reviewing a childhood TB program is crucial to assess how the program is performing to inform decision-making. Childhood TB cases detection performance remained below-set targets as inputs and processes that fed into the program were insufficient. In contrast to our findings, Malik et al. (2019) in Pakistan reported that adequate funding and resources played an important role in improving the performance of TB case detection [17].

Our study revealed that ten tuberculin skin test ampoules expired in 2020 without being requested from the pharmacy. The ten doses that were ordered in March 2020 were not used till the end of 2020. Failure to conduct tuberculin skin tests by health workers could lead to low childhood TB case detection. Our findings are consistent with findings by Schwoebel et al. (2020) in a study in four African countries that reported that health workers had difficulties in administering and reading TST leading to non-use of TST for diagnosis of childhood TB [18].

In this study, the majority of caregivers reported that the children had symptoms of TB, such as cough, fever, weight loss and chest pain for one to 2 weeks before they were diagnosed as TB patients. The delay in TB diagnosis could be attributed to referral from health centres to the district hospital for further investigation such as Chest X-ray which caregivers had to pay for in addition to travel expenses. Similar findings were reported by Pantha et al. (2020) in Bangladesh who noted that TB diagnosis in children was delayed due to further investigations such as magnetic resonance imaging (MRI) which caregivers paid for [9].

Caregivers in our study reported the non-availability of a child-friendly regimen for the 4 fixed-dose combination (FDC) (Isoniazid, Rifampicin, Ethambutol, Pyrazinamide). Similarly, Pantha et al. (2020) in Bangladesh noted that children who needed to take 4FDC had to take 3FDC and isoniazid which was not easy for children to take many tablets leading to poor compliance and poor treatment outcomes [9].

### Limitations

Our study had some limitations. The beneficiaries of the childhood TB program who were interviewed in the study were caregivers of children diagnosed with TB; therefore, children’s perspectives were not explored. We used convenience sampling for the caregivers hence the external validity of the findings presented is low. The study design was descriptive cross-sectional and the participants’ answers were self-reported. Consequently, there may have been a possibility of interviewer bias and social desirability bias. To minimise social desirability bias, the respondents were only given a brief overview of the study from the start. This strategy helps to prevent the respondents from reacting in a particularly socially acceptable manner but creates space to fully explore their views ​​and priorities before concentrating on the main topic of interest. To minimise interviewer bias, we used a well-structured questionnaire for all participants in the comfort of their workplaces.

## Conclusions

The childhood TB program failed to meet its targets due to inadequate inputs namely funds for vehicle and motorcycle servicing, fuel challenges and health care workers with fair knowledge of childhood TB. Apart from that, the district did not manage to perform the planned quarterly TB review meetings, contact tracing and childhood TB training due to lack of funding and COVID-19 lockdown restrictions. No processes, output and outcomes of the childhood TB program were met. Childhood TB case detection remained lower than the national target in 2020 (12%) and needs improvement.

We recommended the integration of sputum specimen transportation with other programs such as the expanded program on immunisation. The district should conduct mentorship and on the job training on the gastric aspirate collection, tuberculin skin test administration, sputum induction, stool collection for Gene Xpert and ongoing advocacy, communication and social mobilization activities to promote childhood TB. The District Medical Officer and the District Nursing Officer to address health workers’ negative attitudes towards gastric aspirates to improve sputum collection.

### Public health actions taken

We discussed the importance of childhood TB with all interviewed health care workers and the results of the study were shared with Chegutu District Health Executive. They agreed to adopt the recommendations and enforce them in the district. We distributed 400 TB notification forms and distributed 400 contact tracing forms to the 34 health facilities.

## Data Availability

The datasets generated and/or analyzed during the current study are not publicly available due to limitations of ethical approval involving the patient data and anonymity but are available from the corresponding author on reasonable request.

## Abbreviations

AIDS: Acquired Immunodeficiency Syndrome
BRTI: Biomedical Research and Training Institute
CDC: Centre for Disease Control and Prevention
CXR: Chest X-ray
DHIS: District Health Information System
DOTS: Directly Observed Treatment Short-course
FDC: Fixed-Dose Combination
HCW: Health Care Worker
MOHCC: Ministry of Health and Child Care
SWOT: Strengths Weaknesses Opportunities and Threats
TB: Tuberculosis
TST: Tuberculin Skin Test
WHO: World Health Organisation
ZAPS: Zimbabwe Assisted Pull System
